# Cold-Resistant Heterotrophic Ammonium and Nitrite-Removing Bacteria Improve Aquaculture Conditions of Rainbow Trout (*Oncorhynchus mykiss*)

**DOI:** 10.1007/s00248-020-01498-6

**Published:** 2020-03-11

**Authors:** Alireza Neissi, Gholamreza Rafiee, Hamid Farahmand, Shadi Rahimi, Ivan Mijakovic

**Affiliations:** 1 Department of Fisheries Sciences, Faculty of Natural Resources, University of Tehran, Karaj, 331585-4314 Iran; 2 Division of Systems & Synthetic Biology, Department of Biology and Biological Engineering, Chalmers University of Technology, Kemivägen 10, 41296 Gothenburg, Sweden; 3 Novo Nordisk Foundation Center for Biosustainability, Technical University of Denmark, 2800 Lyngby, Denmark

**Keywords:** Rainbow trout (*Oncorhynchus mykiss*), Heterotrophic bacteria, Ammonium and nitrite removal, Cold adaptation, Stress, Innate immune response

## Abstract

The aim of this study was isolation and characterization of heterotrophic bacteria capable of ammonium and nitrite removal at 15 °C (optimal temperature for growing rainbow trout *Oncorhynchus mykiss*). Environmental isolates were grown in liquid media containing ammonium or nitrite, and best strains in terms of growth and ammonium or nitrite removal were identified via 16S rRNA sequencing. *Dyadobacter* sp. (no. 68) and *Janthinobacterium* sp. (no. 100) were selected for optimal adaptation to growth at 15 °C and best ammonium and nitrite removal (*P* < 0.05), respectively. A heterotrophic ammonium and nitrite removal (HAN) microbial complex, containing selected strains, was prepared and applied in a trout culture system. After 10 days, the effect of microbial HAN complex was investigated in terms of ammonium and nitrite removal, as well as stress and immune indices present in the plasma of cultivated trout. Compared to a standard cultivation setup, addition of the HAN complex had a clear beneficial effect on keeping the un-ionized ammonia and nitrite level below prescribed standards (*P* < 0.05). This resulted in reduction of stress and immune reactions of cultivated fish (*P* < 0.05), leading to an augmentation of final weight and survival. Application of the selected microbial complex resulted in a significant improvement of the aquaculture ecosystem.

**Electronic supplementary material:**

The online version of this article (10.1007/s00248-020-01498-6) contains supplementary material, which is available to authorized users.

## Introduction

Aquaculture associated to inland water sources is one of the most intensely growing industries, and its growth potential is limited by availability of freshwater sources worldwide [1, 2]. Reusing water is one of the solutions proposed to reduce water consumption in aquaculture. However, the water contains high levels of ammonium, which represents an obstacle for fish breeding [3–5]. Ammonia and nitrite are recognized as the major environmental stressors for fish [6, 7]. Exposure to such stressors results in physiological responses leading weakness in their immune system, which makes them more prone to infections [6, 8] and reduces overall growth and production yield [9].

Attempts have been made to operate freshwater fish farming using some level of water recycling, such as minimal water exchange or recirculating aquaculture systems (RAS) [10, 11]. Nitrification is a microbial process that could be used to reduce or eliminate unwanted nitrogen in recycled water for aquaculture [12–14], and thus make water recycling feasible on a larger scale. Nitrification can be performed in two forms, with autotrophic and heterotrophic bacteria [15, 16], and these bacteria can sometimes work in association [16, 17]. It has been reported that autotrophic ammonium and nitrite removal occurs more rapidly in the presence of heterotrophic strains [18–23].

Rainbow trout (*Oncorhynchus mykiss*) is one of the major cold water species growing worldwide [24]. Trout are generally on-grown in raceways or ponds supplied with flowing water, but some are produced in cages and recirculating aquaculture systems (RAS). In these systems, biofilters based on microorganisms convert harmful components such as ammonium to nitrite and nitrate [25]. The suitable growth temperature for most aerobic heterotrophic species used in biofilters is 28 °C [26], whereas the optimal temperature for trout culture is around 15 °C [17]. Therefore, there is a considerable challenge of finding microorganisms that can grow and efficiently remove ammonium at lower temperatures required for trout aquaculture [27].

The purpose of this study was isolation and characterization of heterotrophic bacteria that remove ammonium and nitrite and can operate at lower temperatures. The aim was to apply such bacteria to rainbow trout culture systems operated at 15 °C, in order to improve environmental conditions in a rainbow trout recirculating aquaculture system and obtain higher production yields.

## Materials and Methods

### Sampling

Three different water sources were collected from different locations in Gothenburg, Sweden, including an artificial lake (57° 41′ 02.6″ N, 11° 56′ 50.0″ E) (SDL), a river (57° 41′ 49.4″ N, 11° 55′ 04.5″ E) (GR), and a natural lake (57° 40′ 42.4″ N, 12° 03′ 25.7″ E, 57° 40′ 42.4″ N, 12° 03′ 25.7″ E) (DL) (Supplemental Fig. 1).

### Isolation of Ammonium and Nitrite Removing Bacteria

Three milliliters of water samples collected from various sources was inoculated in 47 ml of liquid medium adapted for ammonia-oxidizing bacteria (AOB) (containing 279 mg l^−1^ ammonium [28, 29]) and 47 ml of liquid medium adapted for nitrite-oxidizing bacteria (NOB) (containing 427 mg l^−1^ nitrite [30, 31]) and grown in 100-ml sterile flasks at 30 °C. The media were modified by addition of 330 mg l^−1^ glucose and 60 mg l^−1^ peptone as the carbon sources. After 2 weeks, old media were replaced with fresh ones. After 30 days, the cultures were serially diluted up to 10^−8^ and grown on AOB and NOB plates (containing 13 g l^−1^ agar powder, bacteriological). Colonies potentially related to ammonium and nitrite removal were isolated and cultured separately.

### Bacterial Growth and Ammonium or Nitrite Removal Activities

Colonies were grown in liquid AOB and NOB media for 5 days and then subcultured in 96-well plates at 30 °C. Absorbance at 600 nm was monitored every 30 min for 10 days in growth profiler (EnzyScreen Growth Profiler 960). For the selected colonies, ammonium and nitrite concentrations were measured using Hach spectrophotometer DR 3900 (according to the manufacturer’s protocol: LCK340 kits for nitrate assay, LCK303 for ammonium assay and LCK339 for nitrite assay).

### Identification of Bacterial Strains

Genomic DNA of selected bacteria was extracted using DNeasy UltraClean Microbial Kit (Qiagen). The 16S rRNA fragments were amplified using a thermocycler (c1000 touch thermal cycler, BioRad, USA) after preparation with primstar PCR kit, using the following primers: 5′-AGA GTT TGA TCC TGG CTC AG-3′ and 5′-GGT TAC CTT GTT ACG ACT T-3′. The size and quality of 16S rRNA fragments (expected size 1.5 kb) were checked by agarose gel electrophoresis (Supplemental Fig. 2). After a nano-drop quality assay, PCR-amplified 16S rRNA samples were sequenced (Eurofins, Germany) using the same forward and reverse primers that were used for amplification. The forward and reverse sequencing results were assembled using snap gene software and bacterial strains were identified using the blast portal (https://blast.ncbi.nlm.nih.gov/Blast.cgi). A phylogenetic tree was constructed by neighbor-joining method, and reliability of each node was established by bootstrap methods using MEGA4 software.

### Adaptation to Cold Temperature

Following the screening steps, the selected strains were adapted to low temperature. The strains were first grown at 30 °C for 3 days, then at RT (room temperature, 22.3 ± 2.8 °C) for 2 weeks, and finally at 15 °C for 2 weeks.

### Microscopy Slide Preparing of Isolated Bacteria

1.2% agarose was completely dissolved in Tris-HCl (50 mM) by heating. The microscope slides were floated in agarose solution and then 5 μl of bacterial suspension was transferred onto the slides. The slides were covered by cover glass and observed under an optical microscope (× 1000 combined magnification).

### Rainbow Trout Cultivation

The selected and adapted heterotrophic ammonium and nitrite removing bacteria (HAN) were mixed at a ratio of 2.5:1 (ammonium removing bacteria: nitrite removing bacteria) [32] at a dilution of 8.1 × 10^9^ CFU/ml. One liter of HAN mix was transferred to a 400-l tank containing 50 rainbow trout (50.65 ± 3.89 g), at 14.5 ± 1.2 °C (*n* = 3). Negative control group tanks was selected without adding bacteria to the tanks (*n* = 3). HAN (bacterial complex) and negative control tanks were filled with water before the trout were added to each tank. Ammonium, nitrite, and nitrate concentrations were monitored for 10 days. The fish were fed once per day (1% body weight) with fish feed (CP = 38.7 ± 2.8). During the feeding period, water was not replaced, and only water evaporation (1–2% per day) was compensated. Each tank was a recirculating system with a 30 W waterproof pump at the bottom of each tank. Water from the bottom of each tank was pumped through a filter (containing washed sand, glass wool, and 2 cm^2^ sponge particles) at the bottom of the tank and after filtration, it was returned into the tank. For aeration, a central air compressor was connected to each tank via an aquarium water hose connected to ceramic air stones (40 mm × 15 mm).

### Blood Sampling

At the end of the breeding period, after 24 h of starvation, three fish from each experimental unit were randomly sampled. For this purpose, the fish were first anesthetized using benzocaine [33, 34] and then completely dried. Blood sampling from their caudal vein was performed using a 5-ml heparin syringe. Then, a part of each blood sample was centrifuged at 6000 g for 8 min to obtain plasma. The collected sera were stored at − 80 °C until the desired parameters were measured. The remaining blood samples were used for investigation of hematological parameters.

### Immunological and Stress Indicators

Immunological and stress indicators were measured from the obtained sera according to previously described protocols. The assays included quantification of lysozyme as described by Demers and Bayne [35], total protein as described by Nonaka, Iwaki, Nakai, Nozaki, Kaidoh, Natsuume-Sakai, and Takahashi [36], respiratory burst activity as described by Secombes [37], bacterial activity as described by Budiño, Cal, Piazzon, and Lamas [38], complementary activity assay as described by Yano [39], total immunoglobulin as described by Siwicki and Anderson [40], glucose measurement according to Pottinger and Carrick [41], and cortisol assay as described by Pickering, Pottinger, and Sumpter [42].

### Hematological Parameters

Hematocrit, hemoglobin, and number of red blood cells were measured in all experimental groups. Mean corpuscular volume (MCV), mean corpuscular hemoglobin (MCH), and mean corpuscular hemoglobin concentration (MCHC) were measured using the following formulas [43, 44].

$$ {\displaystyle \begin{array}{c}\mathrm{MCV}=\mathrm{Ht}\times \frac{1000}{\mathrm{RBC}}\\ {}\begin{array}{c}\mathrm{MCH}=\mathrm{Hb}\times \frac{10}{\mathrm{RBC}}\\ {}\mathrm{MCH}\mathrm{C}=\frac{\mathrm{Hb}}{\mathrm{Ht}}\end{array}\end{array}} $$

### Statistical Analysis

The normality of data was assessed by the Kolmogorov-Smirnov test. One-way analysis of variance (one-way ANOVA) was used for comparing data means. The significance level between treatments was determined by the Tukey test at 5% level. Statistical analysis was performed by SPSS 17 software in the Windows environment.

### Ethics

Ethical permission for the research obtained from the Ethics Committee of the University of Tehran.

## Results

In order to isolate bacteria that can thrive at low temperature and effectively remove ammonium and nitrite, water samples were collected from three different water sources in Gothenburg, Sweden (Supplemental Fig. 1). Freshwater sources included a river (GR), and a natural (DL) and artificial lake (SDL). After water sampling from different locations, water quality indicators were measured in all collected samples (Table 1). The results showed that different water sources had different concentration of ammonium, nitrite, and nitrate. Among the samples, the DL water contained the lowest ammonium concentration (0.006 mg l^−1^), followed by SDL (0.051 mg l^−1^) and GR (0.135 mg l^−1^) water. Nitrite is the most ephemeral form of nitrogen which is rapidly converted to other products. Nitrite concentrations were similar in all samples.

**Table 1:** Water quality indicators in collected samples from different water sources

Indicator	Water sources		
	GR	SDL	DL
pH	7.3	7.6	7.4
Temperature °C	4°	4°	4°
NH_4_-N (mg l^−1^)	0.135	0.051	0.006
NO_2_-N (mg l^−1^)	0.026	0.024	0.025
NO_3_-N (mg l^−1^)	0.079	0.352	0.053

Artificial lake (SDL), river (GR), and a lake (DL)

### Isolation and Characterization of Ammonium-Removing *Dyadobacter* sp. (no. 68) and Nitrite-Removing *Janthinobacterium* sp. (no. 100)

**Fig. 1:**
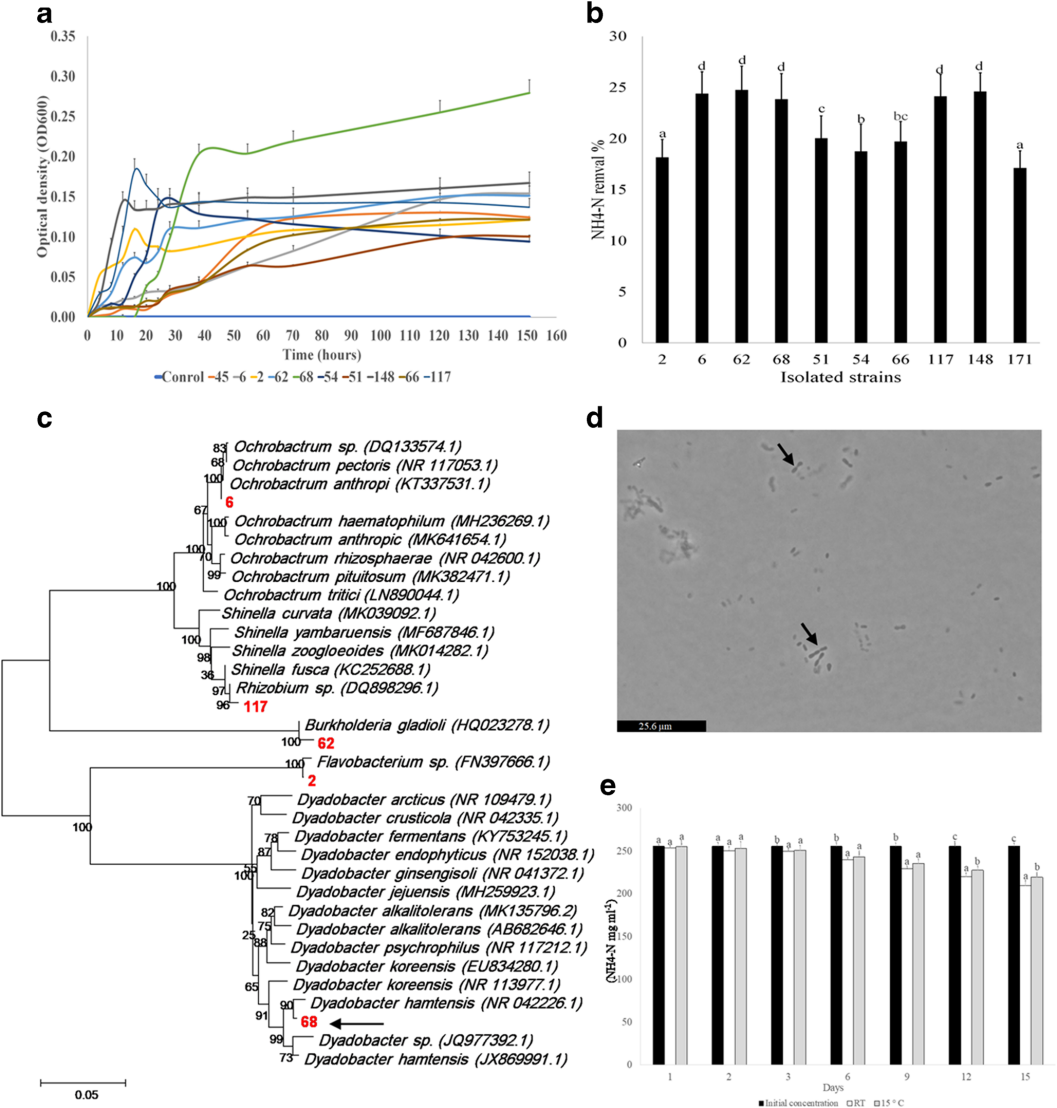
**a** Growth rate of isolated heterotrophic ammonium removing bacteria for 10 days. **b** Ammonium removal activity of selected bacteria after 10 days. **c** Phylogenetic tree of ammonium removing bacteria isolated in this study. **d** Microscopic observation of *Dyadobacter* sp. **e** Ammonium removal activity of *Dyadobacter* sp. at different temperatures

To find a solution for accumulation of unwanted ammonium and nitrite in an aquaculture system, bacterial strains with optimal growth at 15 °C and ammonium and nitrite removal ability were isolated and identified. Among the selected environmental strains with ammonium removal capacity (17.12–24.75% NH4-N removal), samples no. 2 (DL), 6 (SDL), 62 (GR), 68 (DL), and 117 (GR) had superior growth characteristics, with strain no. 68 being clearly the best performer in terms of growth and ammonium removal activity (Fig. 1a, b). Interestingly, strain no. 68 was isolated from the water source collected from DL region with lowest ammonium concentration. Since the use of a new strain in aquaculture system is restricted and one should avoid strains that may infect the fish, the selected strains need to be identified prior to application. Among the isolated heterotrophic ammonium removing bacteria, strains no. 2, 6, 62, 68, and 117 were identified using 16S rRNA sequencing (Supplemental Fig. 3). Figure 1c shows the phylogenetic tree of different heterotrophic ammonium removing species that were isolated in this study. The phylogenetic tree revealed the most closely related relatives of the selected ammonium removing strains. It has shown that the closest evolutionary strain with 94.62% similarity to the best performing strain no. 68 was *Dyadobacter hamtensis* (NR 042226.1) (marked by black arrow in Fig. 1c). Microscopic observation of this strain showed that the cells aggregate in chains which can be helpful for biofloc formation in biofilters and thereby suitable for aquaculture wastewater treatment (Fig. 1d). The isolated strain of *Dyadobacter* sp. (no. 68) is not pathogenic and based on the growth profile and ammonium removal activity, it was selected as the best candidate for further analysis.

**Fig. 2:**
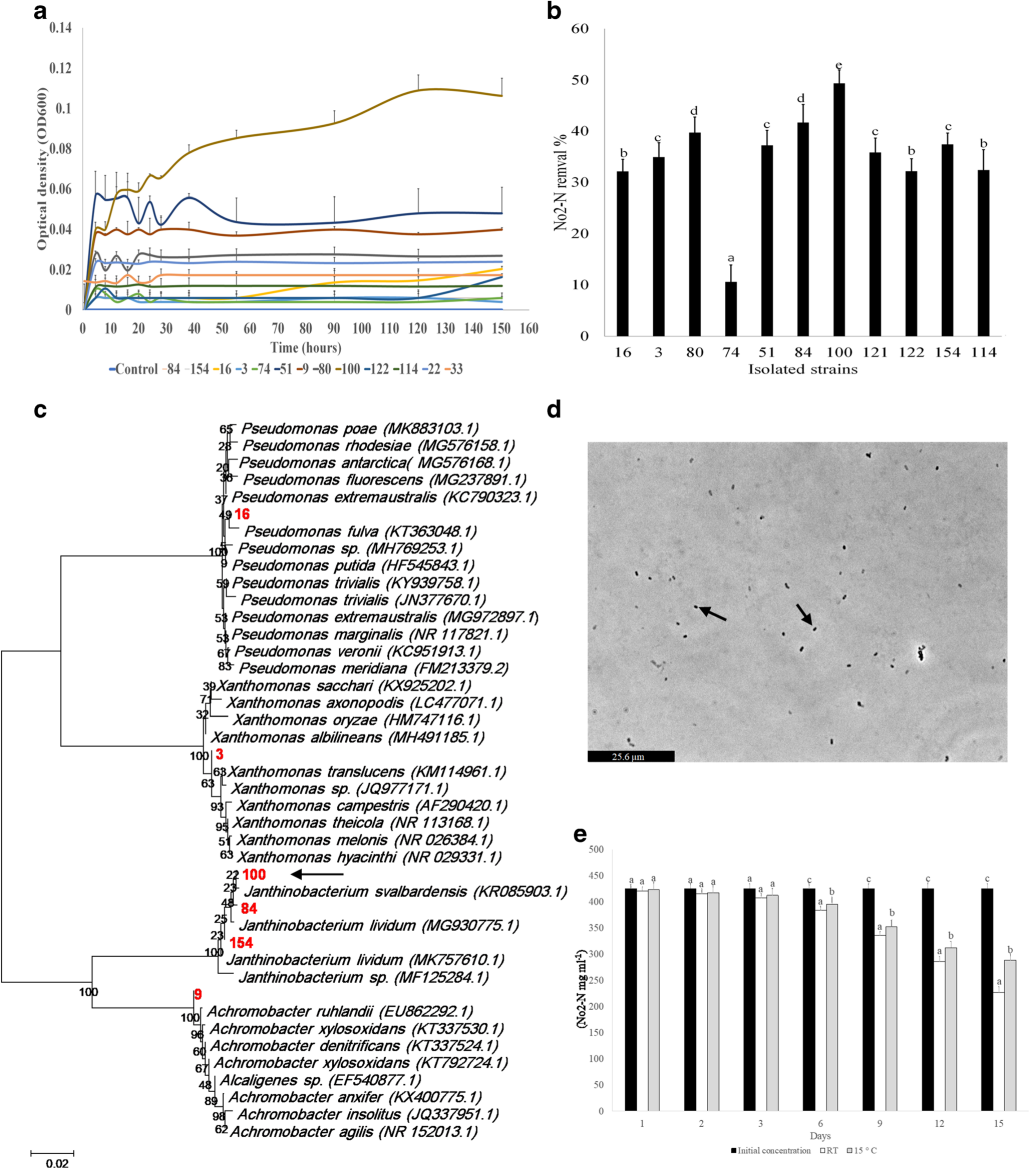
**a** Growth of isolated nitrite removing bacteria for 10 days. **b** Nitrite removal activity in isolated bacteria after 10 days. **c** Phylogenetic tree of nitrite removing bacteria isolated in this study. **d** Microscopic observation of *Janthinobacterium* sp. **e** Nitrite removal activity of *Janthinobacterium* sp. at different temperatures

All selected environmental strains exhibited some capacity to remove nitrite, with their activity ranging from 10.57 to 49.37% nitrite removed from the medium in 10 days (Fig. 2b). Among the selected environmental strains, no. 100 exhibited the best growth and highest nitrite removal activity (Fig. 2a). The group of best performers, among heterotrophic nitrite removing bacteria, strains no. 3 (GR), 9 (SDL), 16 (GR), 84 (SDL), 100 (GR), and 154 (DL) were identified using 16S rRNA sequencing (Supplemental Fig. 4). Figure 2c shows the phylogenetic tree of all heterotrophic nitrite removing species isolated in the current study. Phylogenetic tree showed that among the selected nitrite removing strains, 84, 100, and 154 were close to *Janthinobacterium* genus which can suggest a link between the performance of this genus and nitrite removal. Furthermore, it indicated that the closest strain with 95.33% similarity to strain no. 100 was non-pathogenic *Janthinobacterium svalbardensis* (KR 085903.1) (marked by black arrow in Fig. 2c, D). Therefore, this strain was selected for further analysis as the optimal nitrite remover. It was already reported that the genus Janthinobacterium exhibited an impressive heterotrophic nitrifying efficiency with significant nitrite removal activity [45, 46]. Therefore, it can reduce nitrite to nitric oxide by the nitrite reductase proteins [47].

### Adaptation of *Dyadobacter* sp. and *Janthinobacterium* sp. to 15 °C

Different bacterial strains may not have the ability to adapt and perform certain metabolic processes at low temperatures. On the other hand, rainbow trout is a cold water fish; therefore, the selected strains need to operate at 15 °C in order to be successfully implemented in trout aquaculture. To examine the ammonium removal activity at lower temperatures, the isolated *Dyadobacter* sp. and *Janthinobacterium* sp. were grown at RT (room temperature, 22.3 ± 2.8 °C) and 15 °C. The results showed that *Dyadobacter* sp. retained sufficient ammonium removal activity at lower temperatures (Fig. 2e). The ammonium removal activity commenced around day 6 and continued progressively to day 15. Among the strains isolated in this study, *Dyadobacter* had the highest efficiency of ammonium removal and hence, it was selected for further experiments in trout culturing system. For *Janthinobacterium* sp., the results showed nitrite removal activity at 15 °C and RT starting from day 3. From day 3 until day 9, there was no significant difference in nitrite removal between RT and 15 °C. On days 12 and 15, the highest nitrite removal activity of 11.6 and 13.25 mg l^−1^ day was observed in RT samples, respectively. This investigation indicated that *Janthinobacterium* sp. was able to perform effective nitrite removal at both RT and 15 °C (Fig. 2e) and represents a good candidate for implementation in trout culturing systems.

### Ammonium and Nitrite Levels in Rainbow Trout Culture Were Reduced in the Presence of *Dyadobacter* sp. and *Janthinobacterium* sp.

To determine the ammonium and nitrite removal activity of selected cold-adapted bacteria in trout culture, fresh colonies of *Dyadobacter* sp. (no. 68) and *Janthinobacterium* sp. (no. 100) were used to inoculate AOB and NOB liquid media and grown for 2 weeks, respectively. The culture of *Dyadobacter* sp. (no. 68) and *Janthinobacterium* sp. (no. 100) were applied to the trout breeding system as a mixed culture (*Dyadobacter* sp. (no. 68) to *Janthinobacterium* sp. (no. 100) ratios, 2.5:1) [32]. After 9 days, un-ionized ammonia and nitrite removal activity of the mixed culture was investigated. As expected, the results showed that the un-ionized ammonia concentration in untreated negative control group was above the recommended limits for trout culture. The un-ionized ammonia level in HAN group (8.8 ± 1.8 μg l^−1^) was significantly lower compared to the control (13.2 ± 2.1) (*P* < 0.05). This indicated that *Dyadobacter* sp. was functional in removing un-ionized ammonia in aquaculture system (Table 2) and it is capable of bringing the un-ionized ammonia levels below the recommended limit for trout culture. The results also showed a significant increase of nitrite and nitrate in HAN-treated group compared to the control. In the HAN treatment group, *Dyadobacter* sp. is expected to remove ammonium by converting it to nitrite. As a result, nitrite accumulation could be expected, which is harmful to trout culture [49]. However, nitrite accumulated as a product of *Dyadobacter* sp. activity was effectively removed by *Janthinobacterium* sp. in our HAN microbial complex, resulting in 62 ± 11 μg l^−1^ nitrite concentration in the treatment group which is below the range limit value (1000 μg l^−1^) (Table 2). pH value was lower in the control group in comparison with the HAN group (Table 2). It was previously reported that hydroxamic acids get produced under heterotrophic nitrification [50, 51]. Thus, pH decrease in HAN group might be due to the acid production during nitrification in this experimental group. Other water quality indices such as dissolved oxygen and temperature did not show any significant differences between the groups.

**Table 2:** The effect of heterotrophic ammonium and nitrite removal set (HAN) on concentration of un-ionized ammonia, nitrite, nitrate, dissolved oxygen, pH, and temperature in a rainbow trout (*n* = 50) breeding system after 9 days. Recommended limits according to Timmons and Ebeling [48]

Indicators	Group		
	HAN*	Control	Range limit
NH3-N μg l^−1^	8.8 ± 1.8^a^	13.2 ± 2.1^b^	12. 5
NO2-N μg l^−1^	62 ± 11^b^	31 ± 15^a^	1000
NO3-N mg l^−1^	15.61 ± 5.36^b^	13.13 ± 4.12^a^	< 400
Temperature °C	14.15 ± 1.17	14.13 ± 1.17	< 16
pH	6.85 ± 0.09^b^	6.25 ± 0.17^a^	6.5–8.5
Dissolved oxygen (mg l^−1^)	8.65 ± 2.12	8.51 ± 3.19	> 6

The table shows values of mean ± SD of three experimental repetitions. Values within the same row with different superscript differ significantly (*p* < 0.05)

*Heterotrophic ammonium and nitrite removal set (HAN)

### Trout Stress Was Alleviated, and Aquaculture Productivity Increased in the HAN-Treated Group

Environmental disturbances in aquaculture can lead to increased growth of opportunistic pathogens and decreased appetite of the fish, that negatively affect growth and survival [52, 53]. The trout growth in HAN-treated group increased compared to the control group. The results also showed that the survival rate in the HAN-treated population was higher than in the control group, and the observed differences were statistically significant (Table 3).

**Table 3:** Rainbow trout growth and survival in different experimental groups (HAN and control) in breeding system after 9 days

Group	Indicators		
	Initial weight (g)	Final weight (g)	Survival
Control	50.65 ± 3.89	57.75 ± 3.39^a^	72.25 ± 6.21^a^
HAN*	50.65 ± 3.89	60.75 ± 4.57^b^	86.4 ± 7.29^b^

The table shows values of mean ± SD of three experimental repetitions. Values within the same columns with different superscript differ significantly (*p* < 0.05)

*Heterotrophic ammonium and nitrite removal set (HAN)

Fish gills are responsible for oxygen uptake, and the oxygen is subsequently transported to different fish organs. Any abnormality in fish respiratory system can affect the hematological parameters. Thus, we have investigated these parameters in HAN-treated fish compared to the control group. The results of blood indices showed that hemoglobin, MCV, MCHC%, and hematocrit were higher in the control group compared with the HAN-treated group (Table 4). Red blood cell numbers showed no significant differences between the groups. Increased un-ionized ammonia level can result in fish respiratory disorders in a fish farming environment. Therefore, hemoglobin and hematocrit increase observed in the control group might be correlated with the need to increase of oxygen carrying capacity. However, further investigation of these strains for longer treatment periods and with higher fish density will be required to test this hypothesis.

**Table 4:** Hematological indices (mean corpuscular volume (MCV), mean corpuscular hemoglobin (MCH), and mean corpuscular hemoglobin concentration (MCHC)) in different rainbow trout experimental groups (HAN and control) after 9 days

Group	Indicators				
	Hemoglobin (g/dl)	Hematocrit %	RBC (10^6^ μl^−1^)	MCV (fl)	MCHC%
Control	63.75 ± 2.21	39.5 ± 1.29	1.24 ± 0.03	318.68 ± 12.59	51.41 ± 1.45
HAN*	59.5 ± 1.5	38 ± 1.81	1.25 ± 0.014	304.04 ± 8.23	47.60 ± 1.15

The table shows values of mean ± SD of three experimental repetitions. Values within the same columns with different superscript differ significantly (*p* < 0.05)

*Heterotrophic ammonium and nitrite removal set (HAN)

**Fig. 3:**
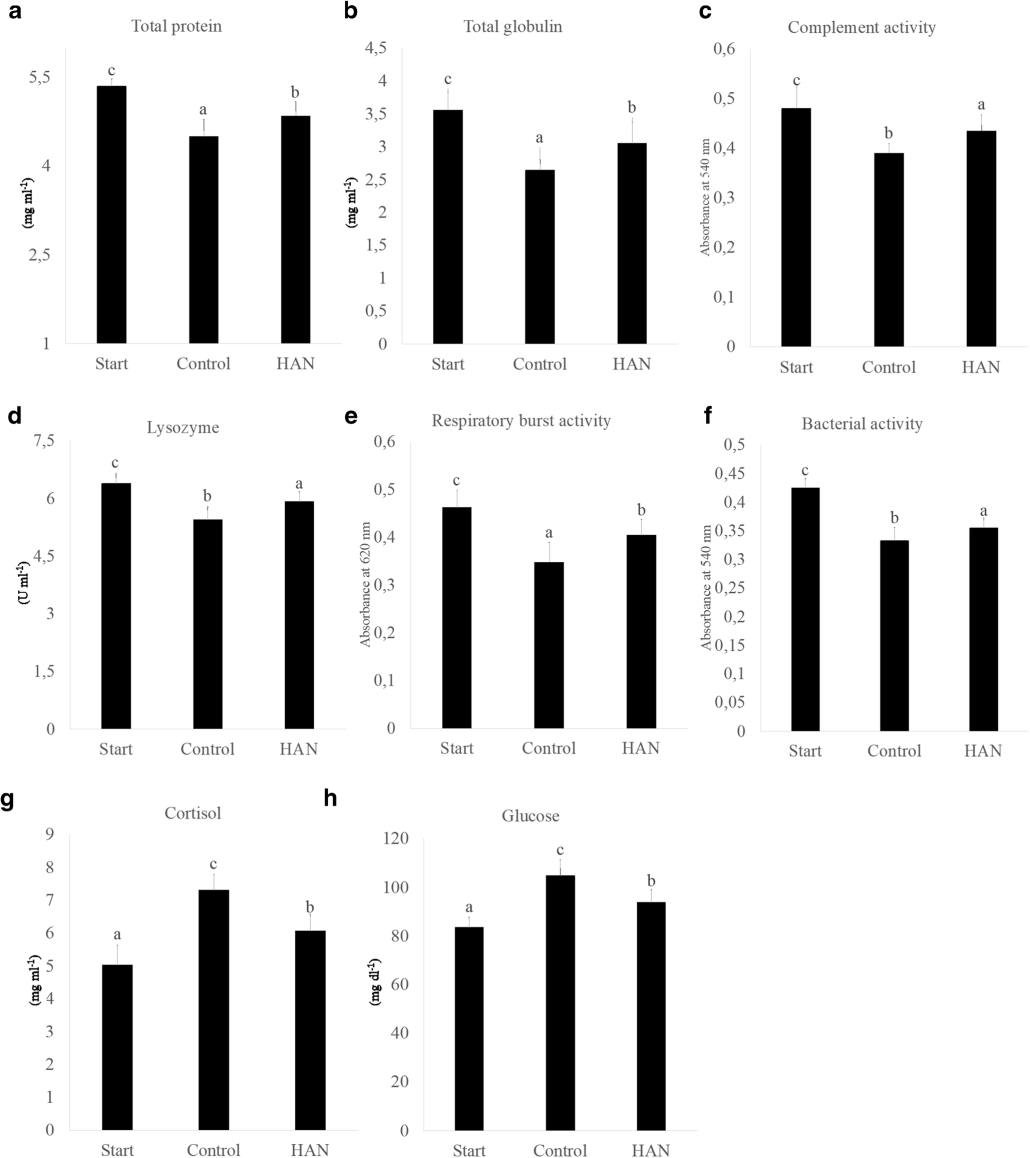
Comparison (mean ± SD) of total protein (**a**), total globulin (**b**), complement activity (**c**), lysozyme (**d**), respiratory burst activity (**e**), bacterial activity (**f**), cortisol (**g**), and glucose (**h**) in rainbow trout sera exposed to microbial complex after 9 days. Start (starting point), control (no microbial complex), and HAN (microbial complex of *Dyadobacter* sp. + *Janthinobacterium* sp.) (*P* < 0.05)

Environmental sources of stress can be broadly divided into acute (short time, high concentration ratio) and chronic (long time, low stress ratio). In a chronic stress, when cortisol level rises, the body energy goes up through the glucose increase, and innate immune is suppressed [54]. Stressors, such as ammonium and nitrite, cause systemic shocks. In this study, the groups were under stress. Our results showed that the glucose and cortisol levels in HAN-treated group were lower than the control at the end of the experiment (Fig. 3), indicating that the fish experienced less environmental stress in HAN-treated group. Growth of opportunistic pathogens also increase in stress conditions [55]. This typically triggers immune system response. Therefore, we have investigated the innate immune response of trout in HAN-treated group compared with the control group. The results showed that the total plasma protein, total globulin, complement activity, lysozyme, respiratory burst activity, and bacterial activity in control group were lower than HAN group at the end of the experiment (Fig. 3), also corroborating the notion that HAN-treated group experienced less stress.

## Discussion

In a RAS system, biological filters are usually used to reduce ammonia. Ammonia is available in two forms, as un-ionized ammonia and ionized ammonium. In terms of toxicity, the un-ionized form is important for aquatic animals [56]. As both groups of heterotrophic and autotrophic bacteria have nitrification ability [56], there is a competition for nitrification between these two groups in a RAS system. Heterotrophic bacteria win this competition for nitrification when dissolved organic carbon (DOC) to nitrogen ratio increases [56, 57]. This ratio could be increased by the activity of heterotrophic bacteria, Accumulation of dead bacteria as well as ammonia assimilation into microbial biomass [57, 58]. It has been reported that by increasing this ratio heterotrophic nitrifiers could have five times higher growth and 2–3 times higher activity compared to autotrophic bacteria [59]. In the current study, heterotrophic *Dyadobacter* sp. and *Janthinobacterium* sp. from environmental isolates were applied for un-ionized ammonia and nitrite removal in trout culture system, respectively.

Prior studies showed that *D. hamtensis* is lipase, maltose, gelatinase and urease negative, glucose, arabinose, galactose and xylose positive, and possesses fermentation ability [60]. The studies have also reported that this species can grow at lower temperatures [60]. It has been previously demonstrated that *Dyadobacter* species can perform denitrification [61]. For example, it was shown that *D. fermentans* and *D. soli* MJ20 are capable of ammonium consumption [62]. In nature, distinct strategies are adopted by microorganisms to cope with nitrogen starvation. *Dyadobacter* can even grow on N-deficient Burk medium due to its ability of N_2_ fixation [63]. However, in our study, ammonium was the only available nitrogen source in the medium for *Dyadobacter* and it was evidently able to grow on it and remove ammonium from the ammonium-enriched medium. Our results suggest that despite a modest decrease of activity at 15 °C, this species can effectively lower ammonium concentration in aquaculture.

The genus *Janthinobacterium* has been isolated from many different water and soil sources so far [64–66]. It has optimal growth at 15 °C and it can also grow at temperatures as low as 2 °C, with a pH of 6.8 [67]. This genus is glucose and oxidase-positive, but indole and lactose negative [67]. Members of this genus are known to degrade nitrite at low temperatures [46]. Several studies report antifungal and antibacterial roles of the *Janthinobacterium* genus [68–71]. These roles are very important in aquaculture systems where opportunistic pathogens are a constant threat.

Some of the major environmental pollutants in trout culture are un-ionized ammonium (ammonia) and nitrite [72]. Ammonia is produced by protein catabolism in fish and excreted from the blood through the gills [73]. A decline in growth, tissue erosion (kidney, gill, and skin) and degeneration, immune suppression and high fish mortality is related to the accumulation of large amounts of ammonium in aquatic systems [74]. The results of the current study showed that selected bacterial complex decreased ammonia in trout culture system. In treated group, decreased mortality and increased growth was achieved. Furthermore, reduced stress and immune reactions in HAN-treated trout in comparison with the control group was observed. Stressors affect the activation of hypothalamus-pituitary-interrenal axis (HPI) in different organs, which is involved in immune and stress response in different species of teleost. Studies showed that although most fishes exhibit a general stress response, the pattern and magnitude of the response may be influenced by environmental factors such as ammonium, temperature, and salinity [75, 76]. Similar to the results of our control setup, cortisol levels were previously found to be increased, and immune responses were decreased in changing or stressful conditions for aquatic species [8, 54, 72].

**Fig. 4:**
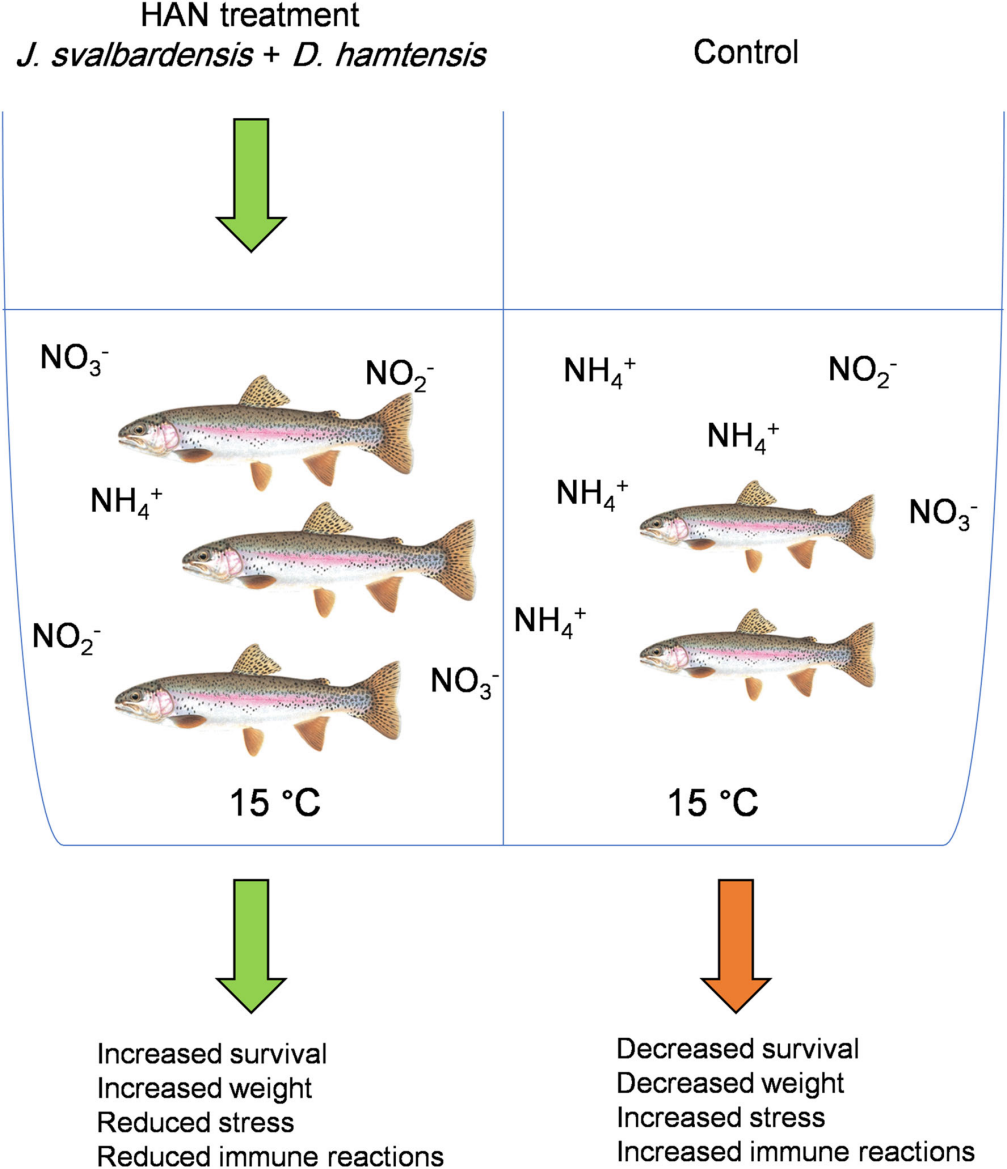
Schematic summary on application of HAN (microbial complex of *Dyadobacter* sp. + *Janthinobacterium* sp.) in on survival, weight, stress, and immune reactions

In the present study, the HAN culture applied to the trout culture system effectively lowered the un-ionized ammonia concentration during trout cultivation. This resulted in improved physiological conditions of trout, with decrease in activation of immune system and enhanced growth (Fig. 4). Nitrite levels were kept in check by *Janthinobacterium* sp., despite the active ammonium to nitrite conversion by *Dyadobacter* sp., and they remained under acceptable limits at all times. Nitrogen immobilization may contribute to nitrogen removal in which microorganisms assimilate inorganic nitrogen such as ammonium and nitrite for the synthesis of proteins and other nitrogen-containing organic compounds [77, 78]. This process occurs frequently when nitrogen-poor organic matter is decomposed [77, 79]. This nitrogen utilization mechanism could be also suggested as a hypothetic mechanism of nitrogen removal using these bacteria. Thus, it can be concluded that the combined use of *Dyadobacter* sp. and *Janthinobacterium* sp. can be recommended for rainbow trout culture systems, as it leads to a considerable improvement of farming conditions. Continued research is recommended by applying longer study period and higher fish density as well as performing further analysis eg additional fish growth indices (SGR, FCR) and oxidative stress analysis. Additionally, further investigation is required to study different mechanism of ammonium and nitrite removal by described heterotrophic microorganisms.

## Electronic Supplementary Material

ESM 1 (DOCX 3427 kb)
