# Supplementary material for: Cold-Resistant Heterotrophic Ammonium and Nitrite-Removing Bacteria Improve Aquaculture Conditions of Rainbow Trout (Oncorhynchus mykiss)
Source: Microb Ecol. 2020 Mar 11;80(2):266–77. doi: 10.1007/s00248-020-01498-6 (PMC7371659; doi:10.1007/s00248-020-01498-6)
Supplement: Supplementary file 1 — (DOCX 3427 kb) [file 248_2020_1498_MOESM1_ESM.docx]

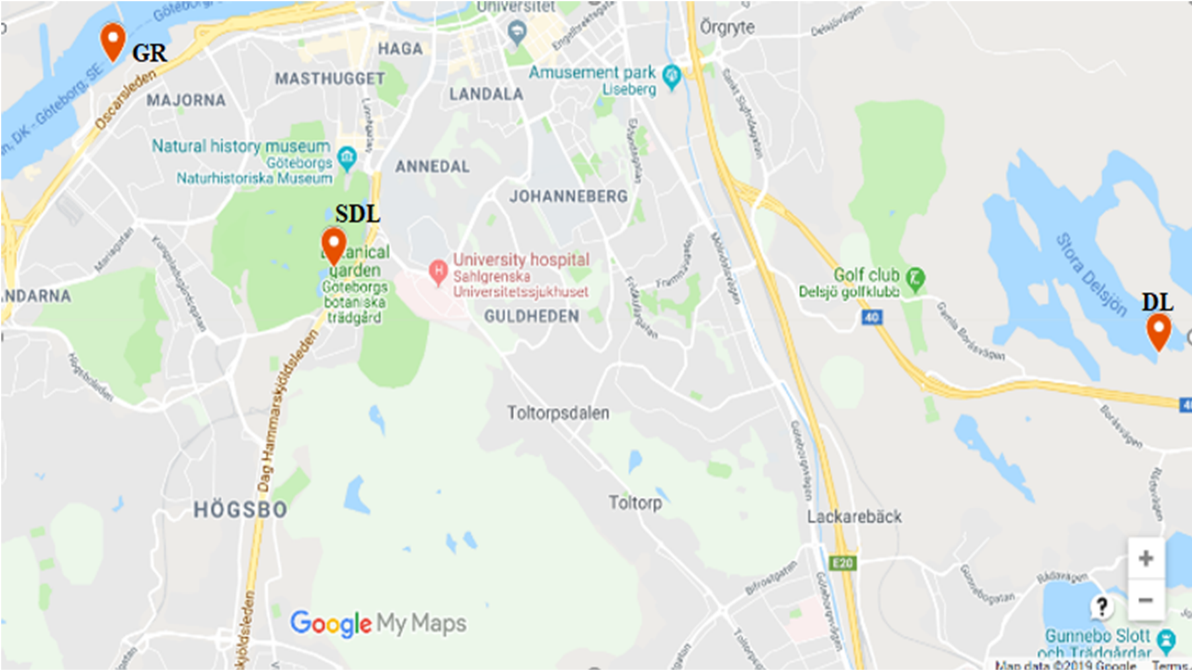


**Supplemental Fig. 1** Sampling spots [artificial lake (SDL), river (GR) and a lake (DL)] in Gothenburg, Sweden (Google Maps).


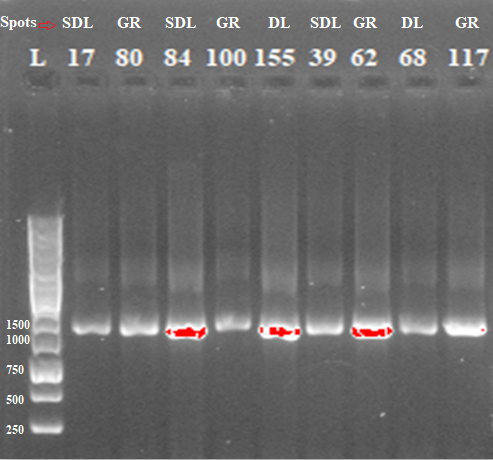


**Supplemental Fig. 2.** 16srRNA fragments isolated from the selected colonies on 1% agarose gel. Ladder was loaded in the first well (L) of electrophoresis gel. Sampling spots marked on top of the gel.

**
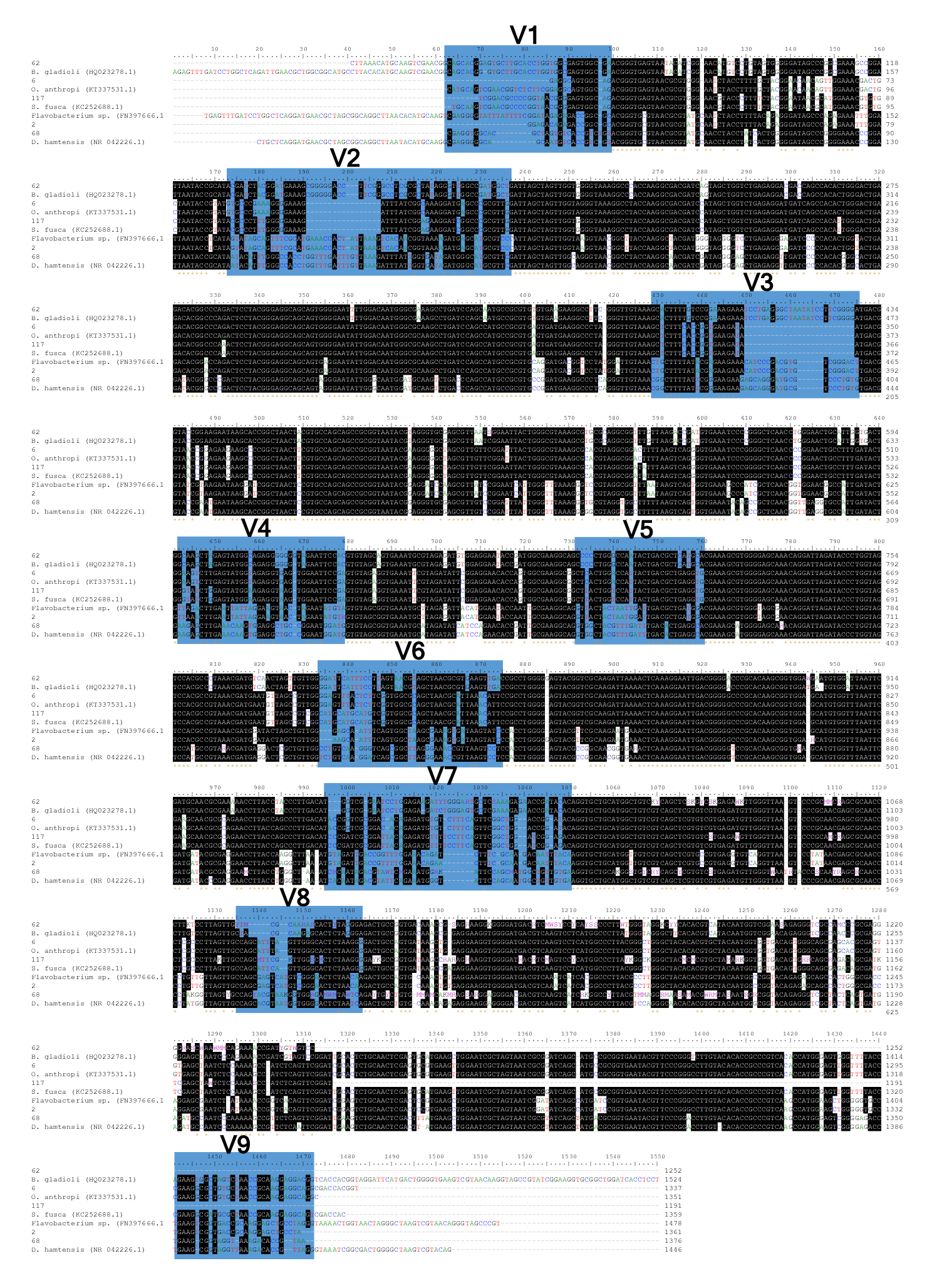
**

**Supplemental Fig. 3.** The variation regions in 16srRNA sequences of heterotrophic nitrite oxidizing bacteria used in this study.


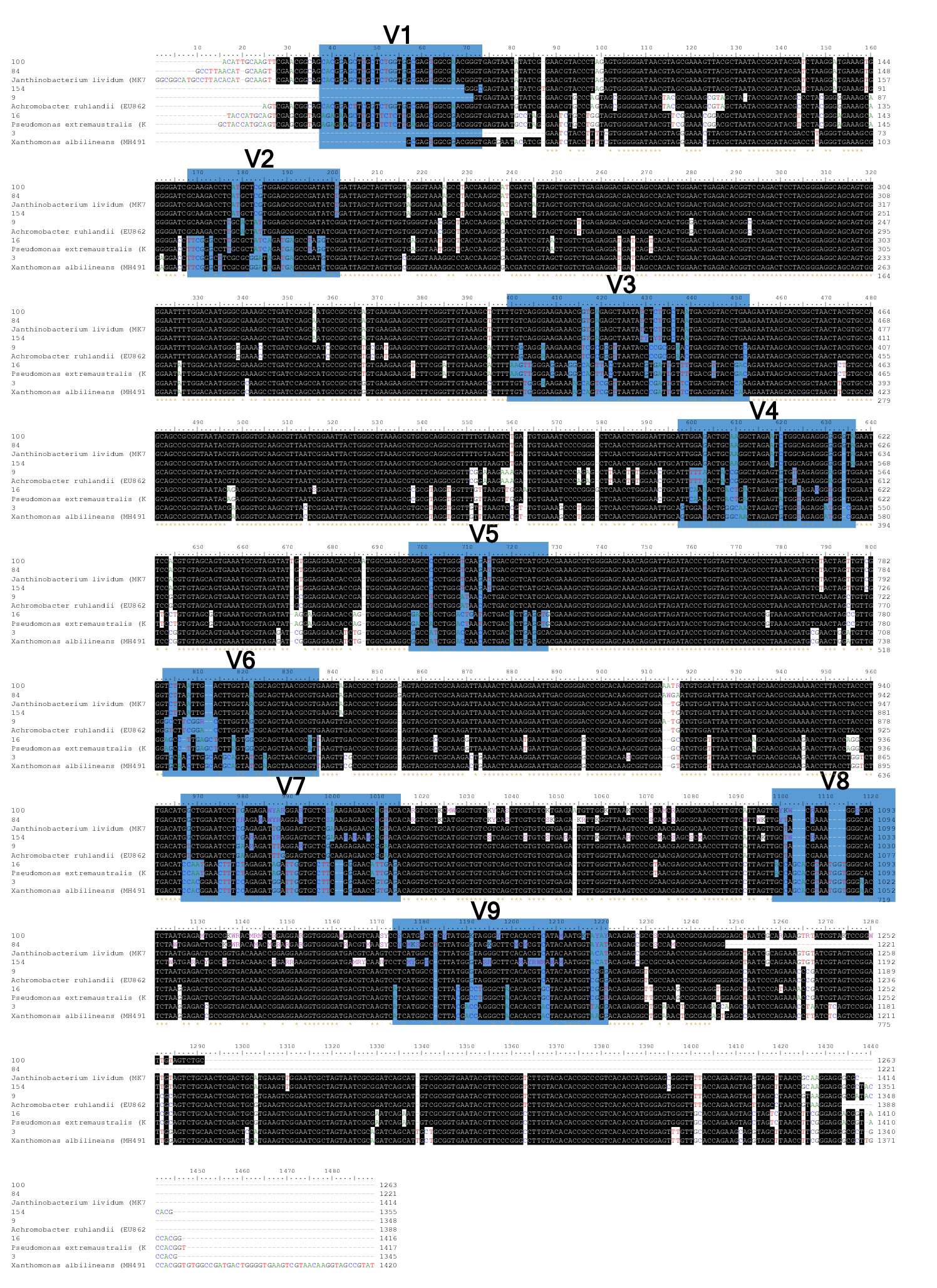


**Supplemental Fig. 4.** The variation regions in 16srRNA sequences of heterotrophic ammonium oxidizing bacteria used in this study.
